# Clinical performance of polyethylenefiber reinforced resin composite restorations in endodontically treated teeth: (a randomized controlled clinical trial)

**DOI:** 10.1186/s12903-024-05009-8

**Published:** 2024-10-24

**Authors:** Ahmed Abdelsattar Metwaly, Amira Farid Elzoghby, Rawda Hesham Abd ElAziz

**Affiliations:** 1 Conservative Dentistry Department, Faculty of Dentistry, Egyptian Russian University, Badr, Egypt; 2 Conservative Dentistry Department, Faculty of Dentistry, Cairo University, Giza, Egypt

**Keywords:** Fiber reinforced composite, Teeth that have undergone endodontic treatment, Ribbond, Clinical trial that is controlled and randomized, Polyethylene fibers, Class II cavities, Bulk fill composite

## Abstract

**Aim:**

The purpose of this study was to evaluate the performance of polyethylene fiber reinforced resin composite fillings compared to bulk fill resin composite fillings in endodontically treated teeth over a two-year monitoring period.

**Method:**

A total of 240 individuals with endodontically treated lower molars and a moderate amount of tooth structure were divided into two equal groups of 120 each. One group received polyethylene fiber reinforced bulk fill resin composite restorations while the other group received only bulk fill resin composite restorations, both applied as per manufacturer guidelines. Two proficient experienced blinded assessors assessed the restorations using modified USPHS criteria at baseline, 6, 12, and 24 months.

**Statistical analysis used:**

Data analysis involved the utilization of Mann-Whitney U, Friedman’s test, and Nemenyi post hoc test, with age data being displayed as mean and standard deviation. The significance level was established as *p* < 0.05, and R software was utilized for statistical analysis.

**Results:**

There were no notable distinctions in any parameters or scores between the intervention and comparator groups at various time points. Alpha scores were present for retention, gross fracture, and secondary caries at all follow up intervals.

**Conclusion:**

Both direct resin composite restorations reinforced with polyethylene fibers and direct bulk fill resin composite restorations placed in endodontically treated molars with moderate remaining tooth structure demonstrated satisfactory clinical outcomes during a 24-month follow-up period.

**Clinical relevance:**

Bulk fill resin composites directly placed in endodontically treated molars with moderate remaining tooth structure showed promise as a treatment option over a two-year period.

**Clinical trial registration:**

(06-01-2022) on https://ClinicalTrials.gov with the ID (NCT05180903).

**Supplementary Information:**

The online version contains supplementary material available at 10.1186/s12903-024-05009-8.

## Introduction

Restoring teeth that have undergone endodontic treatment poses a distinct challenge for clinicians, as these teeth frequently show structural defects caused by decay, prior fillings, and access for root canal therapy. These changes in biomechanics have a detrimental effect on the tooth’s long-term outlook. In the past, it was thought that a root canal filling that was considered clinically and radiographically acceptable could prevent the entry of bacteria and aid in the healing of periapical pathosis. Recent researches have brought into question this idea by presenting proof that emphasizes the importance of the quality of the coronal restoration on tooth stability and resistance to fractures [1]. The current belief is that the main obstacle to leakage is not just well-filled root canals, but also the seal provided by the coronal restoration. Combining the findings from both perspectives; reaching acceptable root canal filling and tooth restoration are crucial objectives for ensuring the attachment apparatus of teeth remains healthy in the 1long run [2, 3]. Current restorative dentistry requires the restoration and the tooth to join together in a way that is mechanically, structurally, and adhesively integrated in order to withstand repeated stress for a long period of time [4]. Dentists encounter difficulties when restoring non-vital teeth, necessitating treatment plans that consider tooth structure, cavity wall thickness, arch position, and applied load [5]. Options for restoring teeth that have undergone root canal treatment involve using crowns, composite resin, and indirect restorations that protect the tooth’s cusps. Resin-based materials improve both fracture resistance and aesthetics, while advanced adhesive systems provide stronger support for tooth structures. Utilizing flowable bulk fill resin composite with a low elastic modulus is recommended as a stress-absorbing layer beneath resin composite restorations to minimize polymerization shrinkage stresses and decrease clinical working time [6].

Composite materials called fiber-reinforced composites (FRCs) consist of three main parts: the matrix (continuous phase), the fibers (dispersed phase), and the interphase within them. FRC materials have high stiffness and strength-to-weight ratios compared to other structural materials, as well as sufficient toughness. FRCs have been utilized in a wide range of engineering and biomedical applications for an extensive period. The literature has discussed the use of short or long fibers to strengthen dental resins for over 40 years. Fiber-reinforced composites made from carbon, polyaramid, polyethylene, and glass have received extensive research and are commonly used in restorative and prosthetic applications [7]. Polyethylene fibers are now being used to enhance the toughness of resin composites, thereby improving their durability and ability to withstand damage.

These reinforcement fibers that can be bonded can be closely fitted to the remaining tooth structure without the need for extra preparation. The polyethylene fiber’s high elasticity modulus and low flexural modulus cause changes in the interfacial stresses along the cavity walls [8]. A systematic review published in 2021 analyzed multiple in vitro studies [9] and concluded that high-molecular-weight polyethylene fibers enhance fracture resistance and promote favorable fractures in teeth that have undergone endodontic treatment. Nonetheless, there is insufficient clinical data concerning this matter. Hence, this randomized clinical trial aimed to assess the clinical effectiveness of polyethylene fiber reinforced resin composite restorations compared to bulk fill resin composite restorations in patients with endodontically treated teeth during two years follow-up period. The proposed hypothesis was null.

## Methods

### Trial design and settings

The experiment was planned as a double-blind trial, with both evaluators and participants were blinded, and was executed as a randomized controlled clinical trial with two parallel groups running simultaneously, each with the same allocation ratio. The trial protocol’s official report can be found on https://ClinicalTrials.gov with the ID NCT05180903 with registration date (January 6, 2022). The study occurred at the Conservative Dentistry clinic of the Faculty of Dentistry at Cairo University, Egypt, from January 2022 to May 2024. The trial was reported in accordance with the guidelines set by CONSORT. Moreover, the Ethics Committee of the Faculty of Dentistry at Cairo University approved this trial, assigning it an ID number of (1/1/22). All individuals received information regarding the goals and processes of the trial. The ethical informed consent was fully explained and clarified and signed by all the participants of the study.

### Eligibility criteria

This study included participants aged between 18 and 55 with proper oral care, who had previously received root canal treatment on molars with Class II cavities with one or two marginal ridges loss and 2 mm wall thickness. The loss of tooth margin at the cervical region was maximum 1 mm below cementoenamel junction and not violating the biological width in all teeth. The study was conducted on 240 class II cavity preparations (120 class II in each group) with loss of two marginal ridges in all teeth for both groups. The chosen tooth should be properly aligned and in regular contact with the nearby teeth.

Patients with underlying medical issues, irreversible pulpitis or necrosis, failed endodontic treatment, lack of opposing teeth, oral habits, severe periodontal diseases or joint issues were excluded from the study [10].

### Determination of the required sample size

A power assessment was carried out to examine whether reinforced composites composed of high molecular weight polyethylene demonstrate a comparable efficacy rate to bulk fill resin composites. Based on the findings from studies [11, 12], the likelihood of achieving score A for the durability and overall integrity of bulk fill resin composite (comparator) was (0.99), while the likelihood of score C was (0.01), with a value of effect size w = 0.98 (*n* = 9). The projected likelihood of staying intact and major breakage for bulk fill resin composite strengthened by high molecular weight polyethylene (treatment) was (0.9) for grade A, (0.1) for grade C with w = 0.8 effect size (*n* = 13). With a significance level set at 0.05 (5%), the power is 80%. The anticipated quantity of samples (n) was a grand total of (200). The sample size was expanded by 20% to compensate for potential dropouts during the follow-up periods, resulting in a grand total of 240 cases, with 120 in each group. The size of the sample was determined using G*Power 3.1.9.4, a well-known software for statistical analysis (https://link.springer.com/article/https://doi.org/10.3758/BF03193146).

### Randomizing, generating sequences, and concealing allocation

Two hundred and forty participants, 115 males and 125 females, with an average age of 31.75 ± 6.33 years in the intervention group (Polyethylene fiber reinforced resin composite) and 32.43 ± 7.00 years in the comparison group (Bulk fill resin composite), were divided into two parallel groups (*n* = 120 each) using an online randomization tool (https://www.random.org/). Random numbers were given out in sealed opaque envelopes. Only the operator was allowed to access this list, and they could only open the envelope when they were ready to apply the composite filling material after finishing the adhesive protocol. Both the two evaluators in charge of assessing trial results and the patients were kept unaware of the specific restorative material used, ensuring a double-blind setup. Blinding the operator was not possible because the techniques needed for applying the various materials varied.

### Approaches: restorative therapy

The same practitioner performed all clinical procedures and was unable to be unaware of the resin composites used because of their different application techniques.

Following evaluation of the root canal procedure, the temporary filling was taken out of the cavity. The cavity was then examined for any carious lesions or undermined enamel that needed to be removed. The teeth were separated using a rubber dam (nic tone natural rubber latex Dental Dam, Zapopan, Jalisco, Mexico) and a stainless steel gauge caliper 0–10 (SALVIN, Germany) was employed to ensure that the thickness of the remaining walls was at least 2 mm. A graduated periodontal probe (Martin, Germany) was used to measure the distance from the cavity floor to the occlusal surface, which was found to be 5–6 mm. A precontoured sectional metal matrix with a ring and saddle (TOR VM, Russia) and a wooden wedge were used to restore the absent proximal walls [10]. Fine Etch^®^ is used for 30 s on the enamel margins and 15 s on dentine when following the Total Etch Technique with 37% phosphoric acid. The etchant was eliminated, and the cavity washed with water spray for 15 s and dried using an air-syringe [11]. The adhesive protocol was identical for all teeth in both groups. Two coats of all-bond universal (Bisco, USA) were applied separately, with the preparation being scrubbed with a micro brush for 10–15 s for each coat. Next, use an air syringe to gently dry the area for a minimum of 10 s [8] before exposing it to a LED light curing unit (Woodpecker Light Cure I Led, China) for 20 s at an intensity of 1600 mW/cm² as per the manufacturer’s guidelines. The radiometer was used to measure light intensity every five patients [10]. Centripetal technique was utilized to restore missing proximal with a 2-mm wedge-shaped universal nano hybrid resin composite (GrandioSO, VOCO) for all teeth. The general nano hybrid resin composite was pressed against the placed matrix band and exposed to the same light curing device for a duration of 10 s. The orifices of the root canals of all teeth were sealed with bulk-fill flowable resin composite (X-tra base, VOCO) and light cured for 10 s.

**For the intervention group**, two pieces of Ribbond fibers were cut according to measures taken at the proximal walls by special scissors (Ribbond Scissors). Next, the fibers were moistened with a resin that was not filled (Ribbond Wetting resin) and then any extra resin was removed with a patient napkin. Next, a 0.5 mm thin layer of Ribbond Securing Composite, a bulk-fill flowable resin composite (X-tra base, VOCO), was placed on the proximal walls. Following this, the dampened and dried parts of Ribbond fibers were promptly placed using the flowable composite as near to the neighboring walls and the pulpal base as feasible and then cured for 10 s. Next, a bulk-fill flowable resin composite (X-tra base, VOCO) was placed in bulk increments around 4 mm thick to fill the cavity, stopping 2 mm before reaching the occlusal surface, and was then cured for 10 s.

**Fig. 1:**
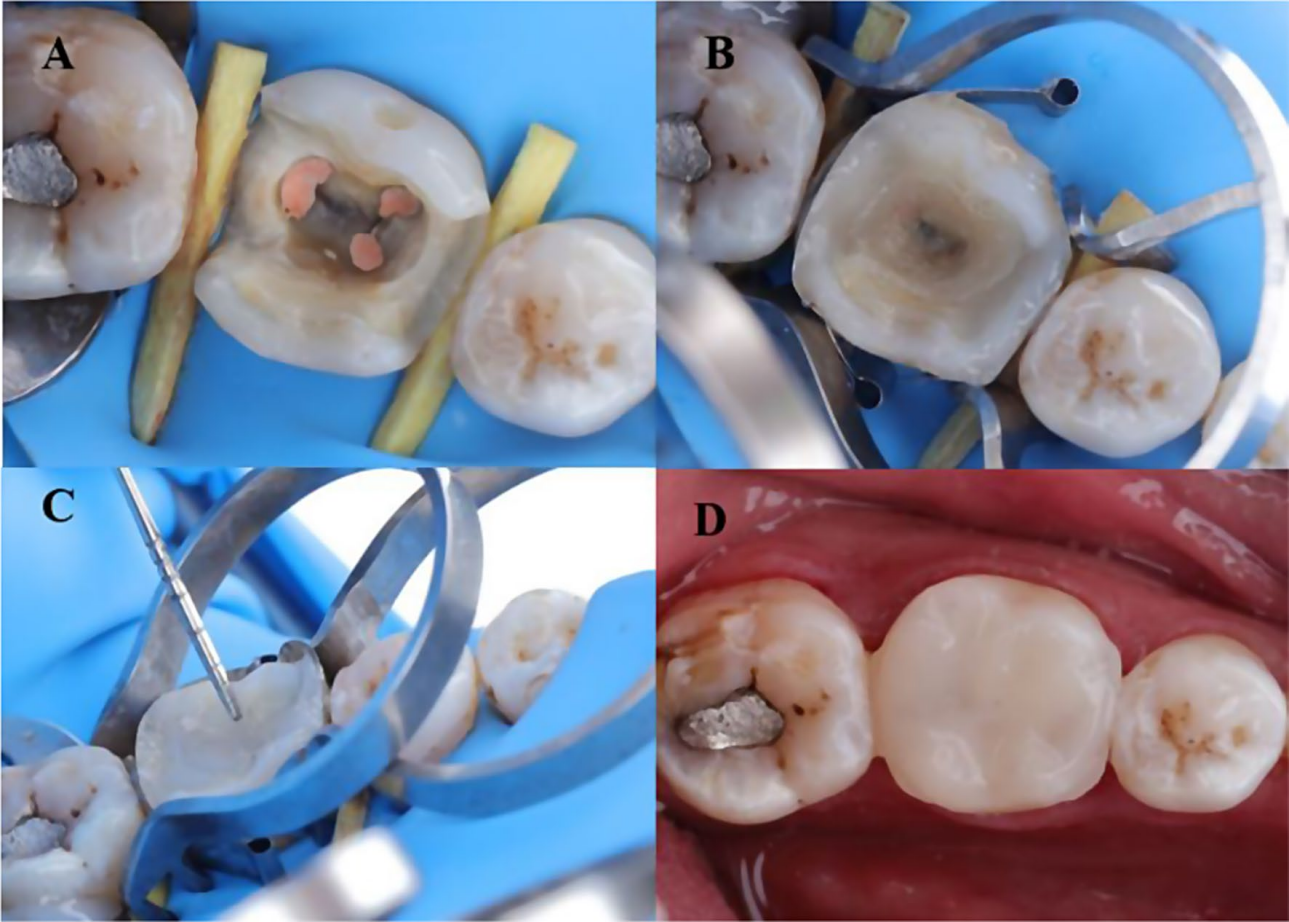
(**A**) Lower first molar cavity has been prepared; (**B**) ribbond pieces were applied against proximal walls; (**C**) the cavity was filled with bulk fill flowable resin composite (X-tra base) but stopped 2 mm before reaching the occlusal surface. Next, the rest of the occlusal portion of the cavity was repaired with a universal nano hybrid resin composite; (**D**) after finishing and polishing of restoration

The leftover top part of the cavity was repaired using a universal nano hybrid resin composite (GrandioSO, VOCO) and then exposed to light for 10 s (Fig. 1). **The comparator group** followed the same procedures for restoring teeth as the intervention group, except they did not use Ribbond pieces at the proximal cavity walls. Finishing was carried out with fine grit yellow coded tapered with round and flame diamond stones by Komet, USA following the evaluation of centric and eccentric occlusion using articulating paper. Polishing was achieved using rubber polishing points from Tangshan UMG Medical Instrument Co., Ltd, China with a low-speed contra-angle handpiece (NACEC, NSK, Japan) to achieve a highly polished surface [10]. All the repair materials were used as per the guidelines provided by the manufacturer (Table 1).

**Table 1:** Material’s specification, composition, manufacturer and lot number

Specification	Material	Composition	Lot number	Manufacturer
Bulk-fill flowable composite	X-tra base	75% weight by weight inorganic fillers in a methacrylate matrix, (Bis-EMA, UDMA)	1,145,403	VOCO, Cuxhaven, Germany
Universal nano hybrid composite	GrandioSO	89% weight by weight inorganic fillers in a methacrylate matrix (Bis-GMA, TEGDMA)	1,215,105	VOCO, Cuxhaven, Germany
Polyethylene fibers	Ribbond	Leno weaved high-molecular-weight polyethylene *(LWHMWPE)* plasma-treated fibers	9002-88-4	Ribbond THM, Ribbond Inc., USA
All-bond Universal BISCO	Universal dental adhesive system	Organophosphate monomer (MDP), Bis-GMA, HEMA, ethanol, water, initiators	B-7202P	Bisco Inc., Schaumburg, IL, USA
Fine Etch^®^	Acid etchant	37% of phosphoric acid gel for etching	FE21159	Spident CO., LTD Namdong-Gu, Korea
Wetting resin	Ribbond Wetting Resin	Mixture of substances with methacrylate ester monomers	800-624-4554	Ribbond THM, Ribbond Inc., USA

*Bis GMA* Bis phenol glycidyl dimethacrylate, *TEGDMA* Triethylene glycol dimethacrylate, *UDMA* Urethanedimethacrylate, *Bis-EMA* bisphenol A ethoxylated methacrylate, *HEMA* 2-hydroxyethyl methacrylate, *MDP* Methacryloyloxydecyl dihydrogen phosphate

### Evaluation of clinical conditions

Two outcome investigators evaluated restoration using modified USPHS criteria in a blinded manner (Table 2). Before beginning the trial, the examiners received training on the adapted USPHS criteria and were required to achieve a kappa value of at least 90% for both inter- and intra-examiner agreement on each criteria. The evaluations of the repairs were carried out at the beginning, 6 months, 12 months, and 24 months time points. Any differences in the scores were addressed through conversation.

**Table 2:** Revised criteria from the United States Public Health Service (USPHS)

Outcome	Criterion	Score	Description	Measuring method
Primary outcome	Retention and Gross Fracture	Alpha	The restoration remains fully preserved, whole, and undamaged.	Mirror inspection visually
		Bravo	A portion of the restoration remains intact and is eligible for repair.	
		Charlie	The restoration is either missing or severely damaged.	
Secondary outcome Secondary outcome	Marginal adaptation	Alpha	The restoration is created to perfectly match the tooth, avoiding any noticeable gaps around the edges that can be felt with a dental explorer.	Examination using a mirror and probe for inspection visually.
		Bravo	The explorer has found a gap in the filling, showing that the filling doesn’t match up perfectly with the tooth’s natural structure. The dentin or base are not visible and the filling is stable.	
		Charlie	The explorer goes into a narrow crevice imperfection that reaches the dentinoenamel junction.	
	Marginal staining	Alpha	There are no visible signs of marginal discoloration that are distinct from the colors of the restorative material and the nearby tooth structure.	Mirror inspection visually
		Bravo	Visual evidence shows slight discoloration at the point where the tooth structure meets the restoration, without extending into the restoration towards the pulp.	
		Charlie	Visual signs reveal slight discoloration where the tooth structure and restoration meet, with the discoloration extending pulpal-wards along the restoration.	
	Secondary caries	Alpha	There is no caries found.	Examination using a mirror and probe for inspection visually.
		Charlie	Caries is found.	

### Analysis based on statistics

Frequency and percentage calculations were used to display both ordinal and categorical data. The chi-square test was used to assess the categorical data. Ordinal data were examined with Mann-Whitney U and Friedman’s test for within-group comparisons, as well as the Nemenyi post hoc test for between-group comparisons. Numerical data was reported using the mean and standard deviation. Once it was confirmed that the age data had a normal distribution, the Shapiro-Wilk test was utilized to assess the normality of the data. Subsequently, the independent t-test was employed for examination. The significance level was determined to be *p* < 0.05 for all tests. R statistical analysis software was used to conduct statistical analysis^2^.

## Results

**Fig. 2:**
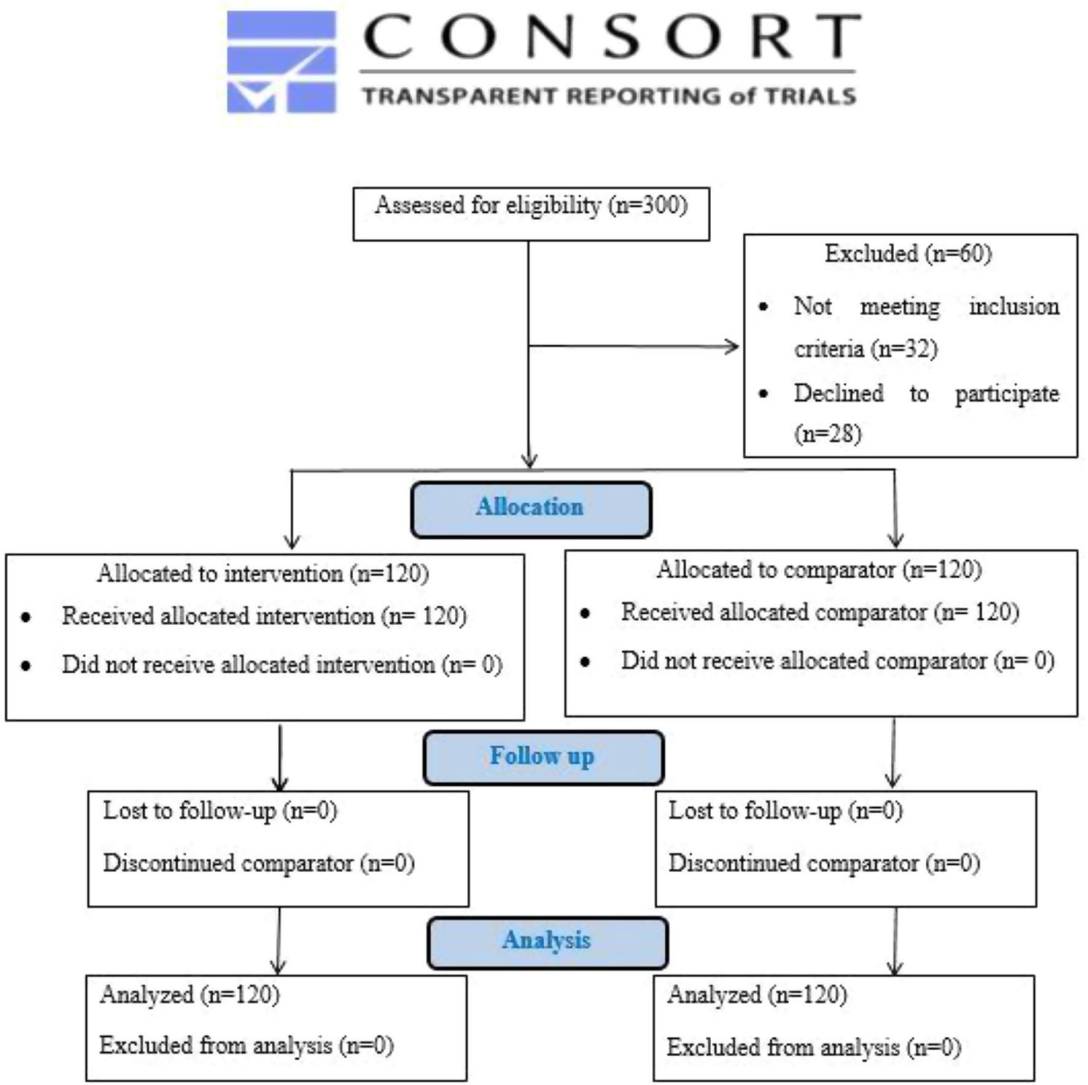
Consort flow diagram showing the process of case selection

The research involved 240 cases that were assigned randomly and equally to each tested group (120 cases per group). All individuals in both the intervention and comparator groups completed the study. This research followed CONSORT guidelines, CONSORT 2010 Flow Diagram and available at (http://www.consort-statement.org), (Fig. 2). In the intervention group, there were 61 males (50.8%) and 59 females (49.2%). In the comparator I group, there were 54 (45.0%) males and 66 (55.0%) females present. The average age of individuals in the intervention group was (31.75 ± 6.33) years, whereas in the comparator group, it was (32.43 ± 7.00) years.

**Fig. 3:**
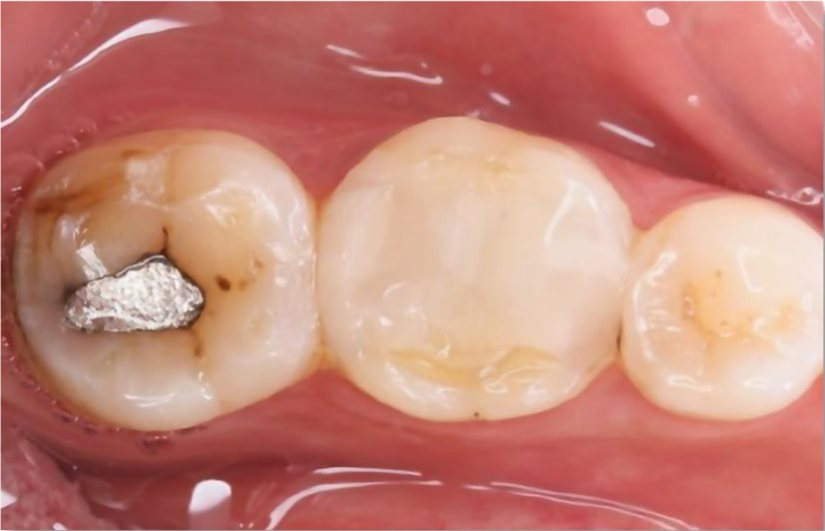
A representative case showing lower first molar restored with Polyethylene Fiber (Ribbond) Reinforced Resin Composite Restoration at 24 months follow up period recorded “Bravo” score in marginal adaptation and staining

**Fig. 4:**
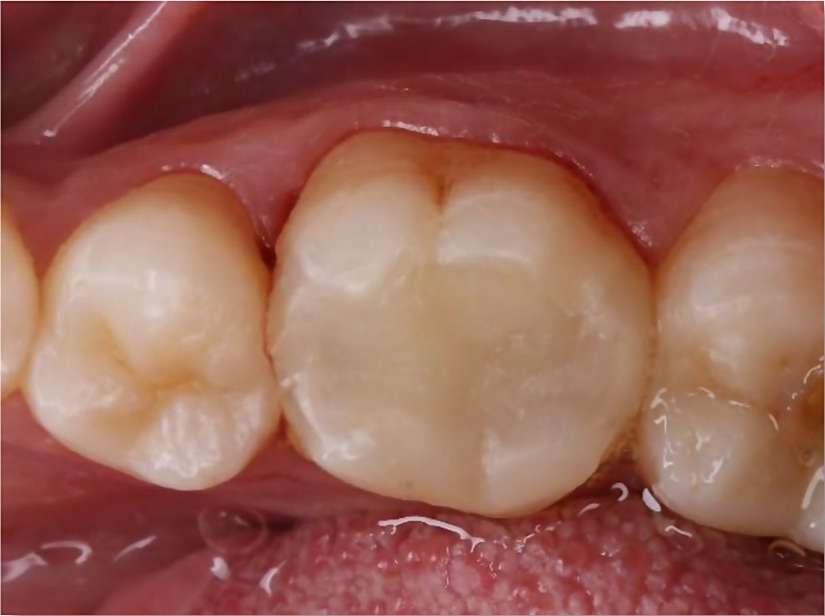
A representative case showing lower first molar restored with Bulk Fill Resin Composite Restoration (X-tra base & GrandioSO) at 24 months follow up period recorded “Alpha” score in marginal adaptation and staining

The majority of the teeth that were treated in both groups were the first molars. Various alterations were observed in terms of marginal adaptation and marginal staining (Figs. 3 and 4). Evaluations were conducted at the start (1 week), 6 months, 12 months, and 24 months. No notable distinction was found among the tested groups in terms of sex (*p* = 0.366), age (*p* = 0.428), and treated tooth (*p* = 0.583). Clinical scores comparisons within and between groups are displayed in (Table 3). All cases in both groups scored an alpha for retention, gross fracture, and secondary caries. There were no significant variations between groups for certain parameters at various time points, but there was a significant difference in scores over time within each group (*p* < 0.05). Post hoc comparisons within each group revealed significant differences in scores between baseline and 6-month measurements compared to later intervals for all other parameters.

**Table 3:** Inter and intragroup comparisons of different clinical parameters

Parameter	Time	Score	*n* (%)		Test statistic	*p*-value
			Intervention	Control		
Retention and gross fracture	*T0*	*Alpha*	120 (100.0%)	120 (100.0%)	**NA**	**NA**
		*Bravo*	0 (0.0%)	0 (0.0%)		
		*Charlie*	0 (0.0%)	0 (0.0%)		
	*T6*	*Alpha*	120 (100.0%)	120 (100.0%)	**NA**	**NA**
		*Bravo*	0 (0.0%)	0 (0.0%)		
		*Charlie*	0 (0.0%)	0 (0.0%)		
	*T12*	*Alpha*	120 (100.0%)	120 (100.0%)	**NA**	**NA**
		*Bravo*	0 (0.0%)	0 (0.0%)		
		*Charlie*	0 (0.0%)	0 (0.0%)		
	*T24*	*Alpha*	120 (100.0%)	120 (100.0%)	**NA**	**NA**
		*Bravo*	0 (0.0%)	0 (0.0%)		
		*Charlie*	0 (0.0%)	0 (0.0%)		
Test statistic			**NA**	**NA**		
p-value			**NA**	**NA**		
Marginal adaptation	*T0*	*Alpha*	120 (100.0%)^A^	120 (100.0%)^A^	**NA**	**NA**
		*Bravo*	0 (0.0%)	0 (0.0%)		
		*Charlie*	0 (0.0%)	0 (0.0%)		
	*T6*	*Alpha*	118 (98.3%)^A^	117 (97.5%)^A^	**7260.00**	**0.655**
		*Bravo*	2 (1.7%)	3 (2.5%)		
		*Charlie*	0 (0.0%)	0 (0.0%)		
	*T12*	*Alpha*	114 (95.0%)^B^	112 (93.3%)^B^	**7320.00**	**0.584**
		*Bravo*	6 (5.0%)	8 (6.7%)		
		*Charlie*	0 (0.0%)	0 (0.0%)		
	*T24*	*Alpha*	112 (93.33%)^B^	110 (91.67%)^B^	**7320.00**	**0.626**
		*Bravo*	8 (6.67%)	10 (8.33%)		
		*Charlie*	0 (0.00%)	0 (0.00%)		
Test statistic			**17.14**	**21.51**		
p-value			< 0.001*	< 0.001*		
Marginal staining	*T0*	*Alpha*	120 (100.0%)^A^	120 (100.0%)^A^	**NA**	**NA**
		*Bravo*	0 (0.0%)	0 (0.0%)		
		*Charlie*	0 (0.0%)	0 (0.0%)		
	*T6*	*Alpha*	118 (98.3%)^A^	117 (97.5%)^A^	**7260.00**	**0.655**
		*Bravo*	2 (1.7%)	3 (2.5%)		
		*Charlie*	0 (0.0%)	0 (0.0%)		
	*T12*	*Alpha*	116 (96.7%)^B^	113 (94.2%)^B^	**7380.00**	**0.357**
		*Bravo*	4 (3.3%)	7 (5.8%)		
		*Charlie*	0 (0.0%)	0 (0.0%)		
	*T24*	*Alpha*	114 (95.00%)^B^	111 (92.50%)^B^	**7380.00**	**0.426**
		*Bravo*	6 (5.00%)	9 (7.50%)		
		*Charlie*	0 (0.00%)	0 (0.00%)		
Test statistic			**12.00**	**18.87**		
p-value			0.007*	< 0.001*		
Secondary caries	*T0*	*Alpha*	120 (100.0%)	120 (100.0%)	**NA**	**NA**
		*Charlie*	0 (0.0%)	0 (0.0%)		
	*T6*	*Alpha*	120 (100.0%)	120 (100.0%)	**NA**	**NA**
		*Charlie*	0 (0.0%)	0 (0.0%)		
	*T12*	*Alpha*	120 (100.0%)	120 (100.0%)	**NA**	**NA**
		*Charlie*	0 (0.0%)	0 (0.0%)		
	*T24*	*Alpha*	120 (100.0%)	120 (100.0%)	**NA**	**NA**
		*Charlie*	0 (0.0%)	0 (0.0%)		
Test statistic			**NA**	**NA**		
p-value			**NA**	**NA**		

*NA* Not Applicable, Values with different superscript letters within the same **vertical column** and **clinical parameter** are significantly different *significant (*p* < 0.05)

## Discussion

An improvement in resin composite technology is focused on enhancing its suitability for posterior teeth with minimal remaining tooth structure by developing fiber-reinforced resin composite materials. These progressions involve both internal and external methods of reinforcement. External reinforcement utilizes Ribbond, a polyethylene fiber-reinforced resin composite, while internal reinforcement uses short fiber-reinforced resin composite (SFRC) with short glass fibers in the filler system. These innovations are designed to enhance the material’s ability to resist crack propagation, thereby strengthening and prolonging the longevity of restorations in compromised teeth [12]. However, a significant challenge in restorative dentistry remains the restoration of endodontically treated teeth. These teeth undergo various changes, including dentin dryness, collagen mutation, and structural loss, which complicate the restoration process [13]. The way these teeth react mechanically is mainly influenced by how much tooth structure is still present. Therefore, preserving as much of the tooth’s natural tissues as possible is crucial [14]. Restoration options for endodontically teeth include direct resin composite, crowns, and posts. While direct resin composite is conservative, it is limited by issues such as polymerization shrinkage, shrinkage stress, and susceptibility to fracture [15]. In contrast, full-coverage crowns, although effective, are invasive and can extend treatment time, potentially reducing the need for further intervention [16].

After endodontic treatment, it is recommended to use reinforcing ferrules in restorations to reduce susceptibility to fractures, particularly when employing complete crowns that cover all cusps [17]. However, the application of posts can weaken roots and increase the risk of perforation during post space preparation [18].

For cases involving minimal to moderate tooth loss and low occlusal forces, direct adhesive resin composite restorations have shown effective results. Studies have shown that there is no notable discrepancy in fracture survival rates between full-coverage crowns and direct resin composite restorations [19]. There is a ribbon reinforcement material called Ribbond which has been on the market since 1992 and comes in different forms with high molecular weight polyethylene fibers. This includes Original Ribbond and Ribbond Triaxial, however, Ribbond THM (Thinner higher modulus) was specifically created with improved flexibility, smoothness, and a higher modulus in mind. The THM version contains a greater amount of thinner fibers (0.18 mm diameter), which is ideal for situations where flexibility and strength are top priorities [20]. Cold-gas plasma treated, pre-impregnated, silanized fibers made of Leno’s high-molecular-weight polyethylene are used to improve the durability and damage tolerance of resin composite in dental restorations by enhancing toughness. These reinforcing fibers that can be bonded easily can conform closely to the remaining tooth structure without the need for extra preparation. The dental composite’s extensive network of threads and secure nodal intersections allows for the redistribution of occlusal forces over a larger surface area. The compact system of secured nodal intersections reduces the risk of fabric damage by stopping fibers from moving during handling and adjustment prior to polymerization. The interfacial stresses on cavity walls in fiber-reinforced restorations are influenced by the high elasticity modulus and low flexural modulus of polyethylene fibers, serving as a protective feature. This is crucial as fractures typically occur above the cementoenamel junction (CEJ), which helps maintain tooth structure and avoid major damage [8, 21].

The significant alterations in tooth biomechanics result from tissue loss due to caries, fractures, or cavity preparation, such as the access cavity created during endodontic treatment. Additional tooth preparation, specifically the removal of marginal ridges, leads to the most significant decrease in tooth stiffness. Studies showed a reduction of 14–44% for occlusal cavity preparations and 20–63% for mesio-occlusodistal (MOD) cavity preparations. In terms of finite element analysis, when utilizing a nonadhesive method (cast gold post and core), the highest stress concentration was observed at the interface between the post and dentin. Conversely, when using fiber-reinforced resin composite posts and cores, stresses increased in the cervical area and had the smallest peak within the root because of a stiffness similar to natural dentin [22]. Various variable factors may impact the link between the survivals of molars that received endodontic treatment. When only looking at the remaining tooth structure, it was discovered that a higher amount of tooth structure led to a greater survival rate.

However, Reports indicated that the survival rates of teeth with MOD resin composite restorations were comparable to those with MO/DO restorations. This showed that the materials chosen for restoring endodontically treated teeth are crucial for the tooth’s lifespan [23].

The study found no significant differences in **retention and gross fracture** scores between the two groups. This could be attributed to the adequate mechanical properties of both polyethylene fiber reinforced resin composites (Ribbond THM) and bulk fill resin composites (X-tra base, GrandioSO), as well as the exclusion of patients with bruxism. These findings align with previous research indicating that bruxism is a significant risk factor influencing the survival of posterior restorations, particularly in terms of restoration fracture [24]. Moreover, the number of surviving walls in the repaired cavities dramatically influenced the survival chances against fracture, confirming the relevance of keeping at least 2 walls, as indicated in this systematic research [19]. Polyethylene fibers are suggested for their stress-modifying impact at the interface between restoration and dentin, potentially decreasing problems such as polymerization shrinkage that could affect restoration outcomes. Prior studies indicate that including a low viscosity intermediate resin can act as a cushion between the bonding agent and composite resin, leading to better outcomes [25].

The study also discovered that there were no significant initial differences in **marginal integrity** scores among the groups, likely due to both composite materials having minimal polymerization shrinkage and shrinkage stress levels. These factors are known predispositions to marginal adaptation failure, as noted in literature [26]. Although both Ribbond THM and X-tra base have potential for improving marginal adaptation, variations in their filler content and coefficient of thermal expansion compared to tooth structure could result in minor differences in adaptation. It is recommended that extended clinical trials are essential to thoroughly evaluate the clinical effectiveness of these materials in the long run [27]. The study’s findings on **marginal staining** indicated no significant difference between the two groups, likely attributable to low polymerization shrinkage and shrinkage stress observed in both types of composites [28]. Marginal discoloration, commonly caused by food and beverage stains and bacterial biofilm, arises from defects between restorations and cavity margins, influenced by factors such as materials used, operator technique, and patient habits [29]. However, conclusive evidence awaits long-term clinical trials. Similarly, regarding **secondary caries**, both groups showed consistent alpha scores throughout the observation period, with no statistically significant differences noted.

This result could be due to the impact of brief monitoring and the primary importance of patient oral hygiene practices and caries risk in deciding the occurrence of secondary caries, regardless of cavosurface margin quality—whether it is exceptional, satisfactory, or diminishing [30, 31]. The study highlighted the importance of strict oral hygiene upkeep and guidance on controlling cavities for all participants, which may have had an impact on the results that were observed.

Based on the study’s findings, both types of resin composites—polyethylene fiber reinforced and bulk fill—prove to be effective choices for restoring molars that have undergone root canal treatment and have moderate remaining tooth structure over a 24-month period. The study found that there was no notable distinction in clinical performance between the two. Therefore, the initial hypothesis was confirmed. The decision between these two materials should be made collaboratively by the patient and dentist, taking various factors into account. Fiber reinforced resin composites like Ribbond demand increased expertise from dental practitioners, additional cooperation from patients, extended time inside the mouth, and entail more procedural stages, resulting in reduced cost efficiency. In contrast, X-tra base and GrandioSO direct bulk fill resin composites need less patient involvement, are simpler to utilize, save time in the mouth, require fewer steps, and are more economical.

## Conclusion

Based on the constraints of the present investigation, it was concluded that both direct polyethylene fiber (Ribbond) reinforced resin composite restorations and direct bulk fill resin composite (X-tra base, GrandioSO) are acceptable to restore endodontically treated molars with moderate amount of remaining tooth structure. However, from the authors’ point of view applying direct polyethylene fiber (Ribbond) reinforced resin composite restorations could be expensive and time consuming in cases of moderate tooth loss.

### Recommendations

1. It is advisable to extend the follow-up period to validate the current findings of this study.
2. Additional randomized clinical trials are suggested to assess the overall effectiveness of fiber reinforced resin composite restorations across different clinical scenarios.

## Electronic supplementary material

Below is the link to the electronic supplementary material.

Supplementary Material 1

## Data Availability

The data sets used and/or analyzed during the current study are available from the corresponding author on reasonable request.

## Abbreviations

FRCs: Fiber-reinforced composites
CONSORT: Consolidated Standards of Reporting Trails
LWHMWPE: Leno weaved high-molecular-weight polyethylene
USPHS: United States Public Health Service
SFRC: Short fiber-reinforced resin composite
THM: Thinner higher modulus
CEJ: Cementoenamel junction

## Footnotes

1 R Core Team (2024). R: An environment and language designed for statistical computing. Vienna, Austria is home to the R Foundation for Statistical Computing. Website address https://www.R-project.org/.

2 R Core Team (2024). R: An environment and language designed for statistical computing. Vienna, Austria is home to the R Foundation for Statistical Computing. Website address https://www.R-project.org/.
